# Unraveling the Web of Delusional Parasitosis: A Case Report

**DOI:** 10.7759/cureus.48550

**Published:** 2023-11-09

**Authors:** Nayan Sinha, Pradeep S Patil, Isha Ahluwalia, Yatika Chadha, Deepa N Sangolkar

**Affiliations:** 1 Psychiatry, Jawaharlal Nehru Medical College, Datta Meghe Institute of Higher Education and Research (Deemed to Be University), Wardha, IND; 2 Psychiatry, Datta Meghe Medical College, Datta Meghe Institute of Higher Education and Research (Deemed to Be University), Nagpur, IND

**Keywords:** iron deficiency anemia delusional parasitosis, delusional parasitosis - clinical, delusional infestations, leprosy and delusional parasitosis, delusional parasitosis

## Abstract

Delusional parasitosis (DP) is a psychiatric condition characterized by the false belief of skin infestation. However, the coexistence of medical conditions, such as iron deficiency anemia, may complicate the clinical presentation and treatment approach. The present case provides an overview of the challenges faced with the diagnosis and management of DP in a patient with a co-morbid medical condition. It highlights the importance of multidisciplinary collaboration in achieving a favorable outcome. Through this case, we shed light on the possible multifactorial origins of DP and emphasize the importance of a comprehensive approach to care for patients with this condition. It also underscores the need for timely recognition and appropriate treatment to improve the quality of life for individuals affected by this debilitating psychiatric condition.

## Introduction

Delusional parasitosis (DP) is characterized by a "persistent state in which the individual firmly believes that tiny creatures like insects, lice, pests, or maggots are residing and flourishing either on or inside their skin, even in the face of substantial evidence to the contrary" [1]. The condition typically manifests in individuals aged 45 years or older, often occurring after middle age, and some experts suggest that it is more commonly observed in females [2].

Two forms of DP have been identified. Primary DP is not associated with any underlying disorder. It meets the criteria for persistent delusional disorder or delusional disorder somatic type [3]. In contrast, a secondary form of the condition emerges in conjunction with other psychiatric conditions like substance abuse, depression, dementia, schizophrenia, etc. It is common for DP to occur simultaneously with various physical ailments such as renal insufficiency, multiple sclerosis, diabetes mellitus, hepatitis, vitamin B12 deficiency, iron deficiency anemia, or leprosy [4-5]. DP can also be triggered by medications, such as amantadine, anticholinergic drugs, levodopa, or, in rare cases, certain antibiotics [6].

The clinical presentation exhibited by the patient in this disorder is characteristic. The primary symptom of utmost significance is the patient's report of sensations resembling the presence of crawling, biting, or nesting insects. Typically, individuals provide highly detailed accounts of the parasites' characteristics and behaviors, and many invest substantial amounts of time, finances, and energy to eliminate these perceived insects. One noteworthy observation is that patients present samples of suspected pathogens to the medical professional as evidence supporting their belief that their skin, various body parts, or immediate surroundings are affected by infestation in “matchboxes” or sealed containers. This phenomenon has been described as the “matchbox sign” in various studies [7-8]. Many patients with DP experience a feeling of being misunderstood, which can result in social isolation and the emergence of depressive symptoms [9]. This case highlights a unique interplay between DP and its complex multifactorial origins.

## Case presentation

A 65-year-old married man, belonging to a low socio-economic status background, with a well-adjusted premorbid personality, presented with a three-year history of intense itching and uncomfortable sensation of insects crawling all over his body. He brought several self-collected samples of the “insects” in sealed containers, which were found to be skin debris and lint. He had sought consultations with multiple dermatologists, but a comprehensive skin examination had revealed nothing.

His belief in insects crawling all over his body was firmly held despite multiple reassurances and evidence. He was highly distressed by his condition and had intermittent depressive symptoms in the form of low mood, loss of energy, loss of interest in pleasurable activities, and passive death wishes. There was trouble initiating and maintaining sleep because of the unpleasant sensations, and he frequently skipped his meals because he felt as if the insects were crawling inside his mouth and over his food.

The patient had a past history of untreated leprosy at the age of 11. There were minute nodules (palpable, solid lesions that are greater than 10mm) all over his limbs. No deformity was present during that time. History of leprosy treatment at the age of 21 for which the patient was admitted. No deformity of limbs and joints was present. He again presented with leprosy at 60, for which he was admitted to a tertiary care hospital for a month and given medications for a year. He acquired deformity of limbs and nose at that age. There is also a history of vision loss in both eyes for the last two years. The patient had no other prior medical or psychiatric history before this current presentation.

The patient took treatment for his "parasitic infestation" from the Dermatology and Psychiatric Department of various hospitals, but no improvement was noticed. Unfortunately, his treatment records were unavailable. He was admitted to the Psychiatry Department of our hospital with the above-mentioned complaints. 

A multi-disciplinary approach was employed for his management. Initially, the patient underwent a dermatological examination to rule out any genuine signs of infestation. The general physical assessment showed itch marks on the skin of all limbs, pallor in both eyes, and bilateral pedal edema as the only notable findings. After confirming the absence of actual infestation, the psychiatric team explored the patient's unshakable belief that he was infested with insects. We inquired whether he would consider any alternative explanation for his skin sensations. The patient adamantly rejected any alternative explanation, even when presented with evidence in the form of collected samples that revealed no insects. Simultaneously, routine medical tests were conducted, revealing a low hemoglobin level of 6.3%, consistent with the findings from the general physical examination. A consultation with a medical specialist confirmed a diagnosis of iron deficiency anemia, and a blood transfusion was recommended by the medical team. During the mental status examination, significant findings included a depressed affect, delusion of parasitosis in his thought content, and tactile hallucinations affecting his perception. While under observation in the ward, his behavior included hallucinatory actions of picking his skin in an attempt to catch the imagined insects.

A diagnosis of secondary DP was considered. The primary focus of the treatment process was to establish a strong and positive connection with the patient. He was given reassurance that his distress was acknowledged as genuine, and a neutral approach was adopted in relation to his belief about being infested by insects in order to avoid reinforcing the delusion. Additionally, he received psychoeducation about the disorder and its different potential causes for insight facilitation.

He was prescribed moisturizing lotion by the dermatologist for his itching. The psychiatric team prescribed a 5mg hora somni (HS) (at bedtime) dose of olanzapine, which was gradually increased to 10mg HS to manage his delusional symptoms. However, minimal improvement in his mental status examination and insight was observed within 10 days. Following this, the patient received blood transfusions with packed red blood cells and was also given oral iron supplementation over a span of two weeks. His hemoglobin levels improved gradually, after which, significant improvement in his symptoms was noticed. The patient no longer complained of itching, and his sleep and appetite significantly improved. His mental status examination at the end of one month showed no abnormalities.

## Discussion

In a suspected case of DP, where patients hold an unwavering belief in being infested with insects despite contradictory evidence, a thorough diagnostic evaluation is essential when selecting the appropriate treatment for a condition with multiple potential causes. It is crucial to consistently eliminate the possibility of a real parasitic infestation before arriving at this diagnosis. After excluding actual parasitosis, it becomes imperative to investigate various secondary factors, such as systemic diseases, other psychiatric disorders, or medications, which could have triggered or played a role in these abnormal sensations. This assessment helps determine the specific type of DP [9].

Given the potentially diverse origins of this disorder, it is essential to take into account all potential causes and subsequently choose a suitable treatment for the particular variant of DP afflicting the patient. When enduring symptoms like persistent delusions are present, it always warrants the use of antipsychotic medications [10]. Presently, first-line treatments primarily involve second-generation antipsychotic medications, mainly due to their improved safety profiles and greater tolerability [9]. In the comprehensive literature review conducted by Freudenmann and Lepping [11], it was observed that risperidone and olanzapine were the most frequently employed atypical antipsychotic medications [11]. For elderly patients, it is necessary to make dosage adjustments based on their age, as well as considering their cardiac, kidney, and liver functions. Typically, dosages can range from 1mg to 8mg per day for risperidone and from 5mg to 10mg per day for olanzapine [12]. An additional option could be aripiprazole with a dose ranging from 2mg to 10mg per day, known for its minimal side effects and lack of weight gain. Nevertheless, it's worth noting that its effectiveness in DP has been described in only seven case reports [13,14].

The bulk of treatment research has predominantly cantered around antipsychotic medications. However, some case reports have indicated varying degrees of benefit with serotonergic antidepressants. It is recommended for individuals whose delusions may have an underlying cause related to depression, anxiety, or obsessive-compulsive disorder. It's worth noting that individuals with milder delusions or greater insight into their condition have been suggested as more appropriate candidates for antidepressant therapy [15].

A clinical response typically begins to manifest around 1.5 to 2 weeks after initiating pharmacotherapy, with the maximum effect often reached within six weeks. It's important to note that discontinuing therapy after achieving a clinical response frequently leads to symptom relapse [13].

However, the treatment will not be very effective if the patient has some underlying physical ailments. For example, treatment with antipsychotics did not show the desired improvement level usually observed in the case of primary DP in our patient. A swift improvement in his condition became evident upon the correction of his hemoglobin levels. In another study conducted by Sekhon et al. [5], it was concluded that the patient’s DP resolved completely with the correction of iron deficiency anemia [5].

Another causative factor of DP could be leprosy. It has been reported that in individuals with leprosy, early-stage symptoms typically include localized sensations like tingling, reduced sensitivity, shooting pains, and itching as noted by Jopling and Harman in 1986 [16]. It is proposed that the symptoms appear to have sparked the onset of the DP. One theory posits that these patients experience significant impairment in their capacity to differentiate between typical and unusual bodily sensations and the delusion might be influenced by internal dysfunction within the limbic system [16-17]. Our patient had a previous medical history of leprosy, which could be a contributing factor to the development of DP in conjunction with iron deficiency anemia.

This present case highlights that for effective diagnosis and treatment of DP a strong collaborative effort involving dermatologists, psychiatrists, and physicians is required.

## Conclusions

DP is a challenging condition that often requires a coordinated effort among mental health professionals, dermatologists, and primary care physicians. The presentation of this case underscores the importance of thorough evaluation to rule out organic causes and the need for a multidisciplinary approach to managing this rare but debilitating condition. This case report demonstrates the importance of a comprehensive evaluation and highlights the potential benefit of addressing medical comorbidities, such as iron deficiency anemia, in managing DP. Nonetheless, our study does possess some constraints. For instance, the patient was not granted a longer observation period that is, two weeks, to assess the impact of only the antipsychotic medication. In response to the patient's distress and the absence of any improvement with antipsychotics within the initial ten days, a decision was made to administer a blood transfusion to promptly address the low hemoglobin levels and get faster results. Further research is warranted to explore the relationship between anemia and psychiatric symptoms in such cases.
